# Supplementary material for: Staphylococcal Enterotoxin M Exhibits Thrombin-like Enzymatic Activity
Source: Biomolecules. 2025 Sep 24;15(10):1357. doi: 10.3390/biom15101357 (PMC12564132; doi:10.3390/biom15101357)
Supplement: Supplementary file 1 [file biomolecules-15-01357-s001.zip › biomolecules-3629684-supplementary figures.pdf]

Report of N-terminal sequencing

Name of the sample: TWF0815

Customer name: Ji Liu

Client: Southwest Minzu University

Testing agency: Sangon Biotech (Shanghai) Co., Ltd.

Testing personnel: Ming Zhang

Results and analysis:

a) Calibration of standard samples: Calibration of 19 kinds of PTH-amino acids mixture.

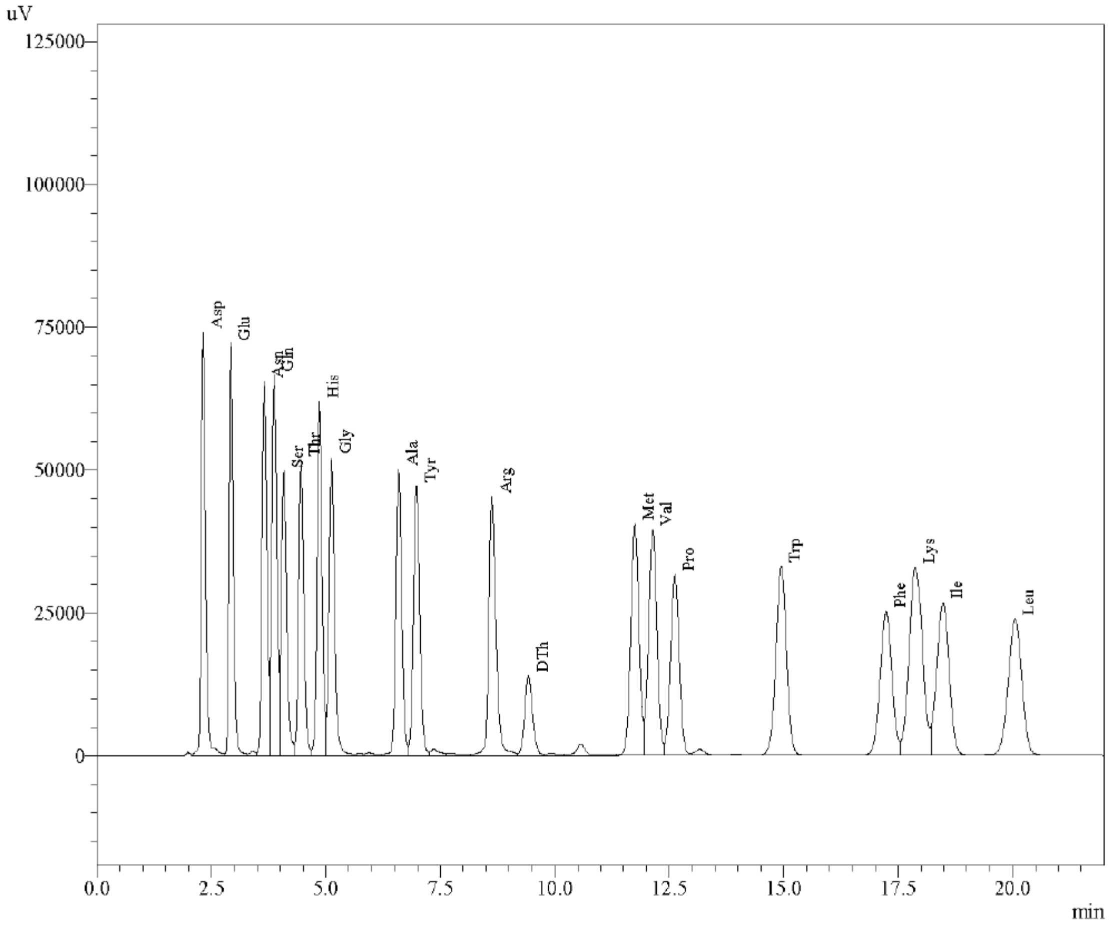

Figure S1. Chromatogram of calibration of 19 kinds of PTH-amino acids mixture.

b) customer sample

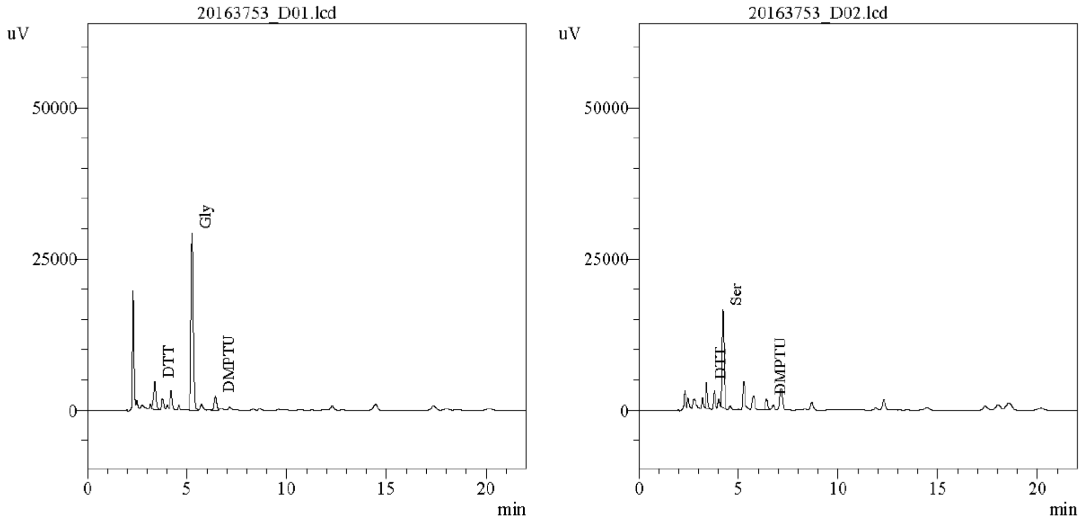

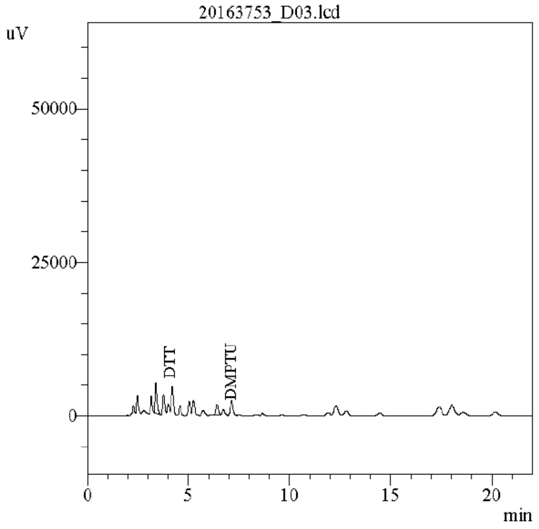

20163753_D03.lcd
uV
50000
25000
0
DTT
DMPTU
0
5
10
15
20
min

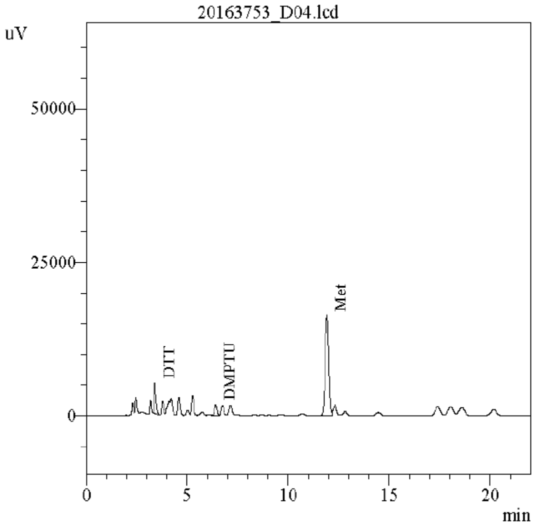

20163753_D04.lcd
uV
50000
25000
0
DTT
DMPTU
Met
0
5
10
15
20
min

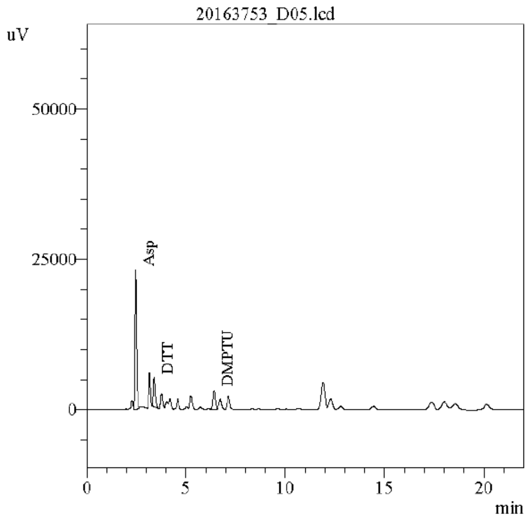

20163753_D05.lcd
uV
50000
25000
0
Asp
DTT
DMPTU
0
5
10
15
20
min

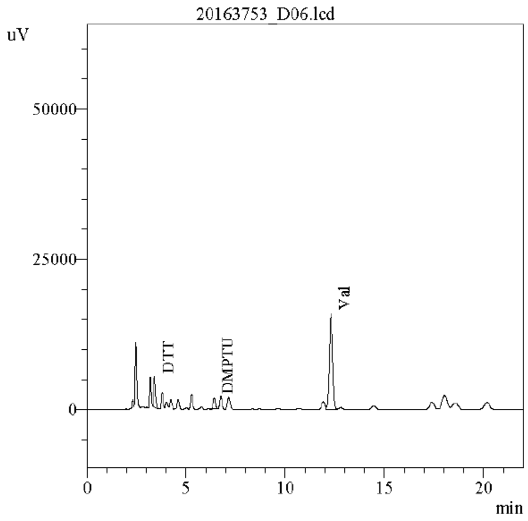

20163753_D06.lcd
uV
50000
25000
0
DTT
DMPTU
Val
0
5
10
15
20
min

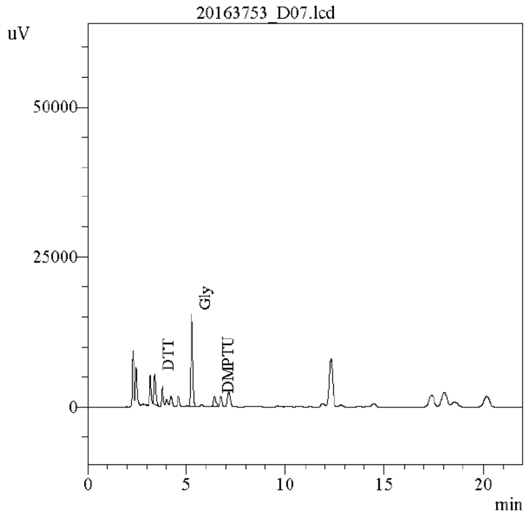

20163753_D07.lcd
uV
50000
25000
0
DTT
Gly
DMPTU
0
5
10
15
20
min

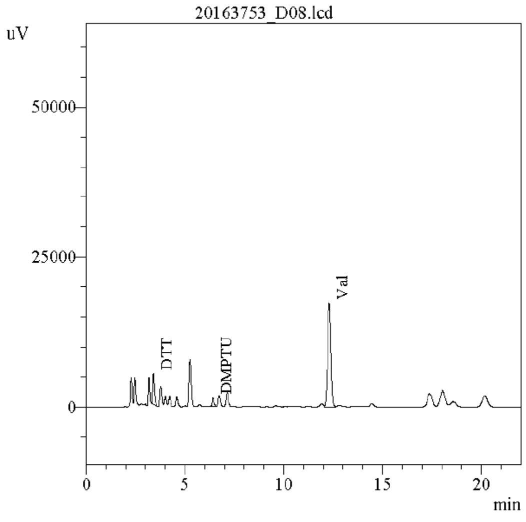

20163753_D08.lcd
uV
50000
25000
0
DTT
DMPTU
Val
0
5
10
15
20
min

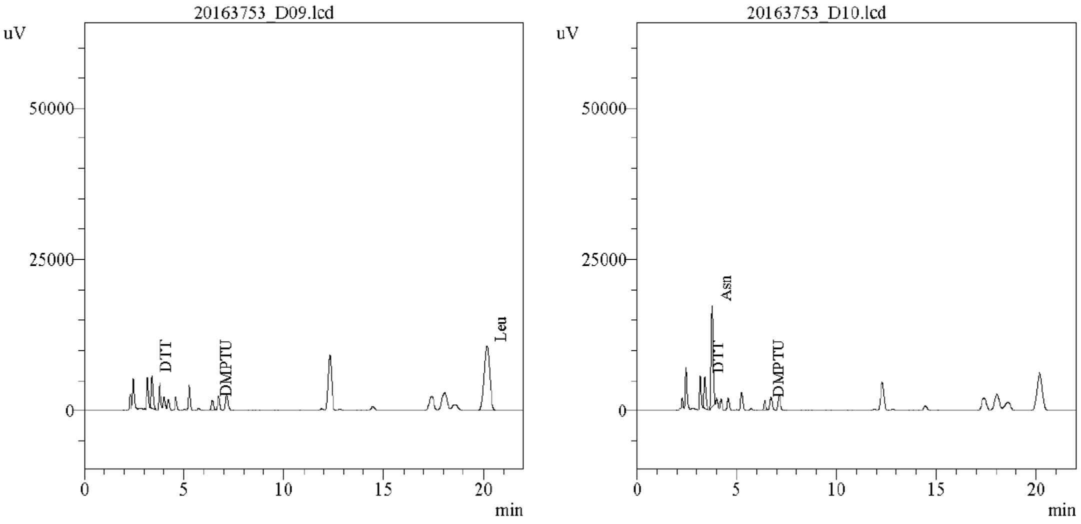

Figure S2. N-terminal sequencing chromatogram of customer sample (TWF0815)

Conclusion:

N-terminal sequence of sample named 15-193 (TWF0815) was determined as NH2-Gly-Ser-X-Met-Asp-Val-Gly-Val-Leu-Asn.

Remarks: the "X" used to mean Cys.
