# Supplementary figures and images for: Staphylococcal Enterotoxin M Exhibits Thrombin-like Enzymatic Activity
Source: Biomolecules. 2025 Sep 24;15(10):1357. doi: 10.3390/biom15101357 (PMC12564132; doi:10.3390/biom15101357)

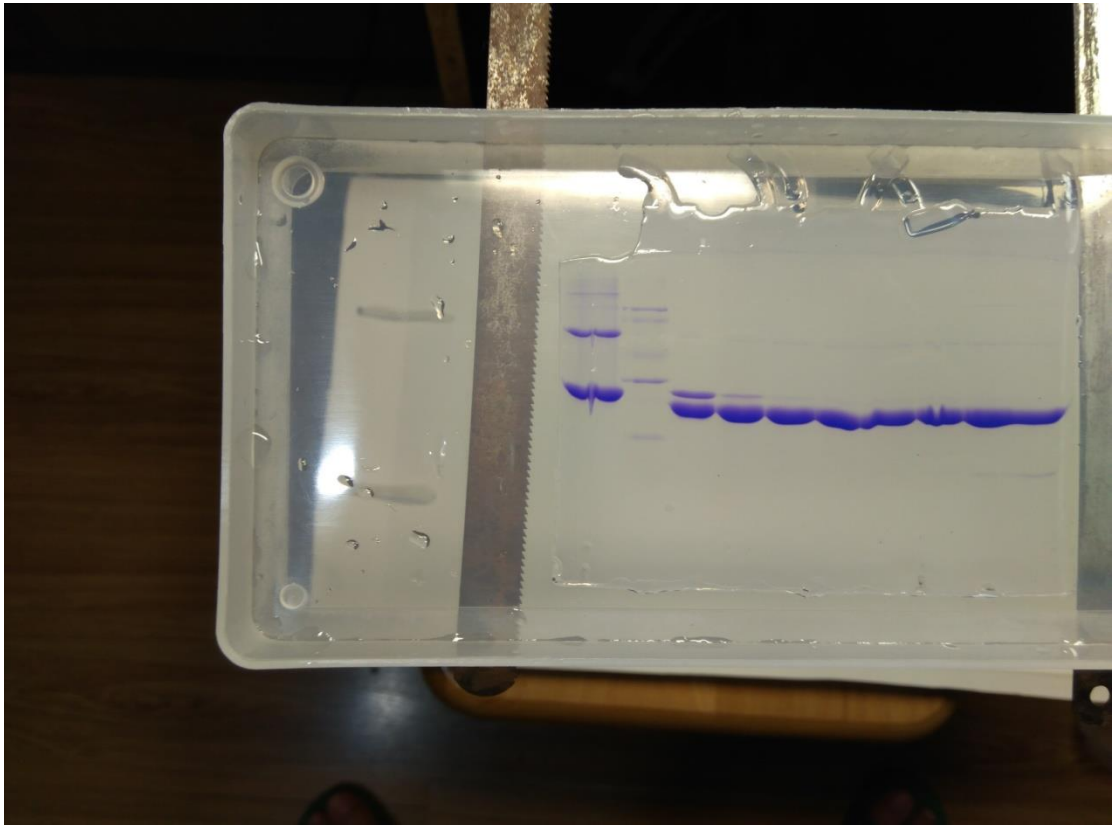

Figure 1 (b)

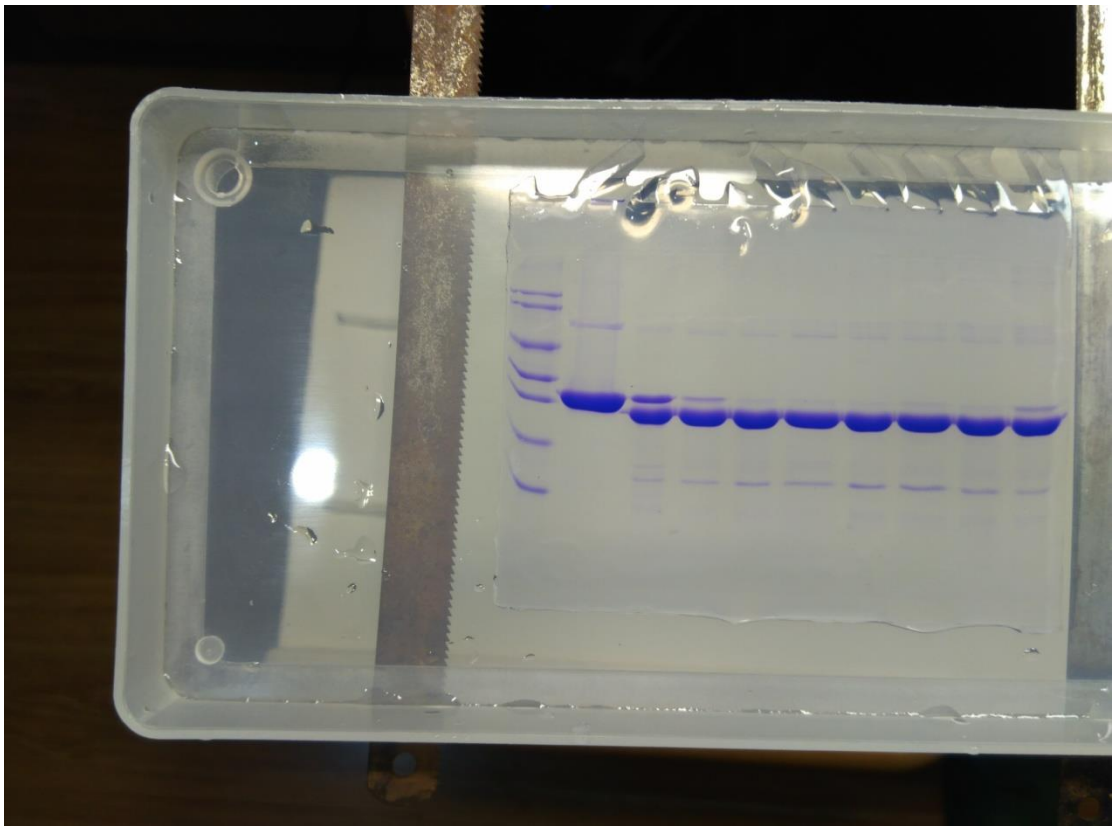

Figure 1 (c)

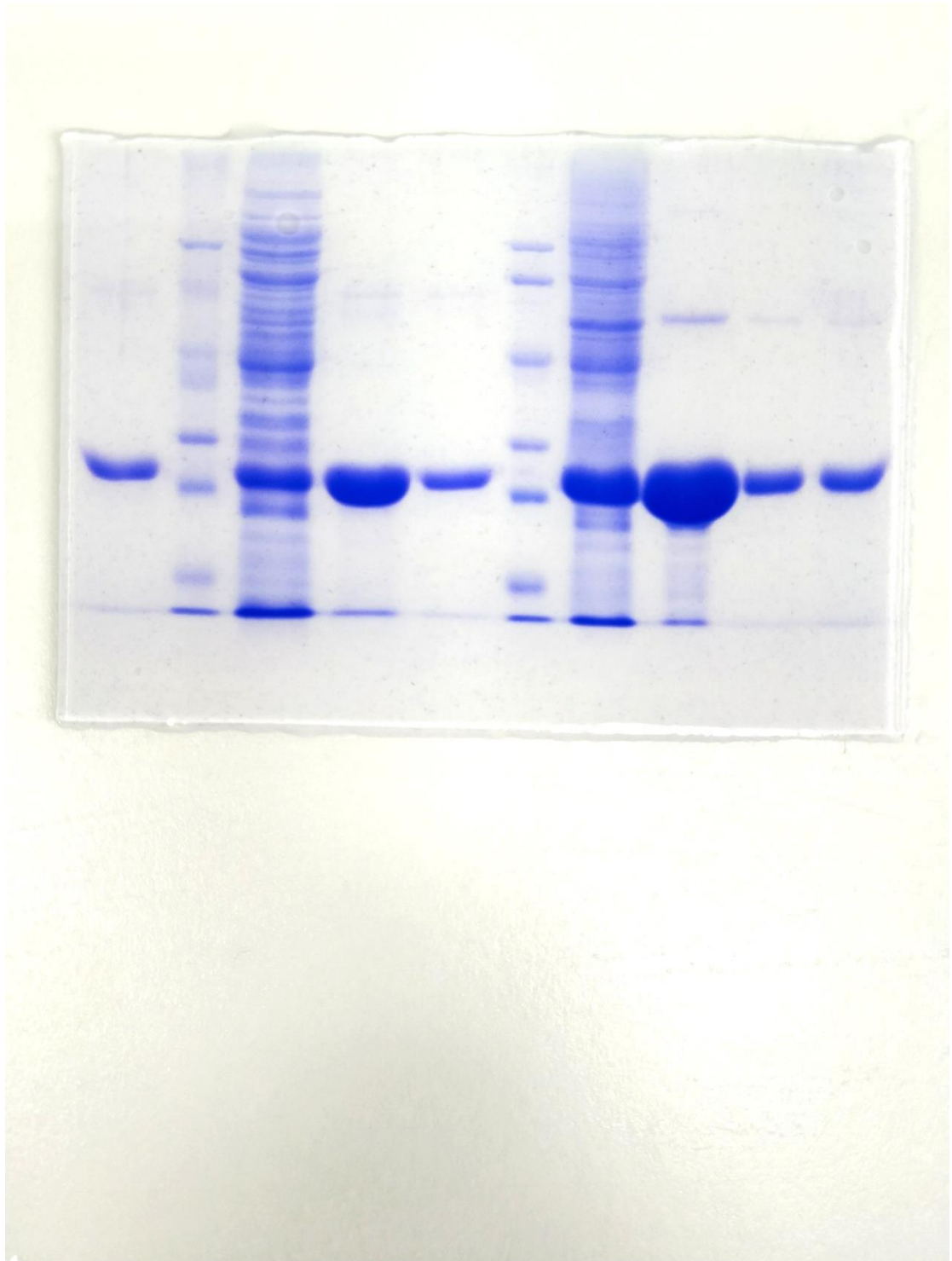

Figure 2 (f)

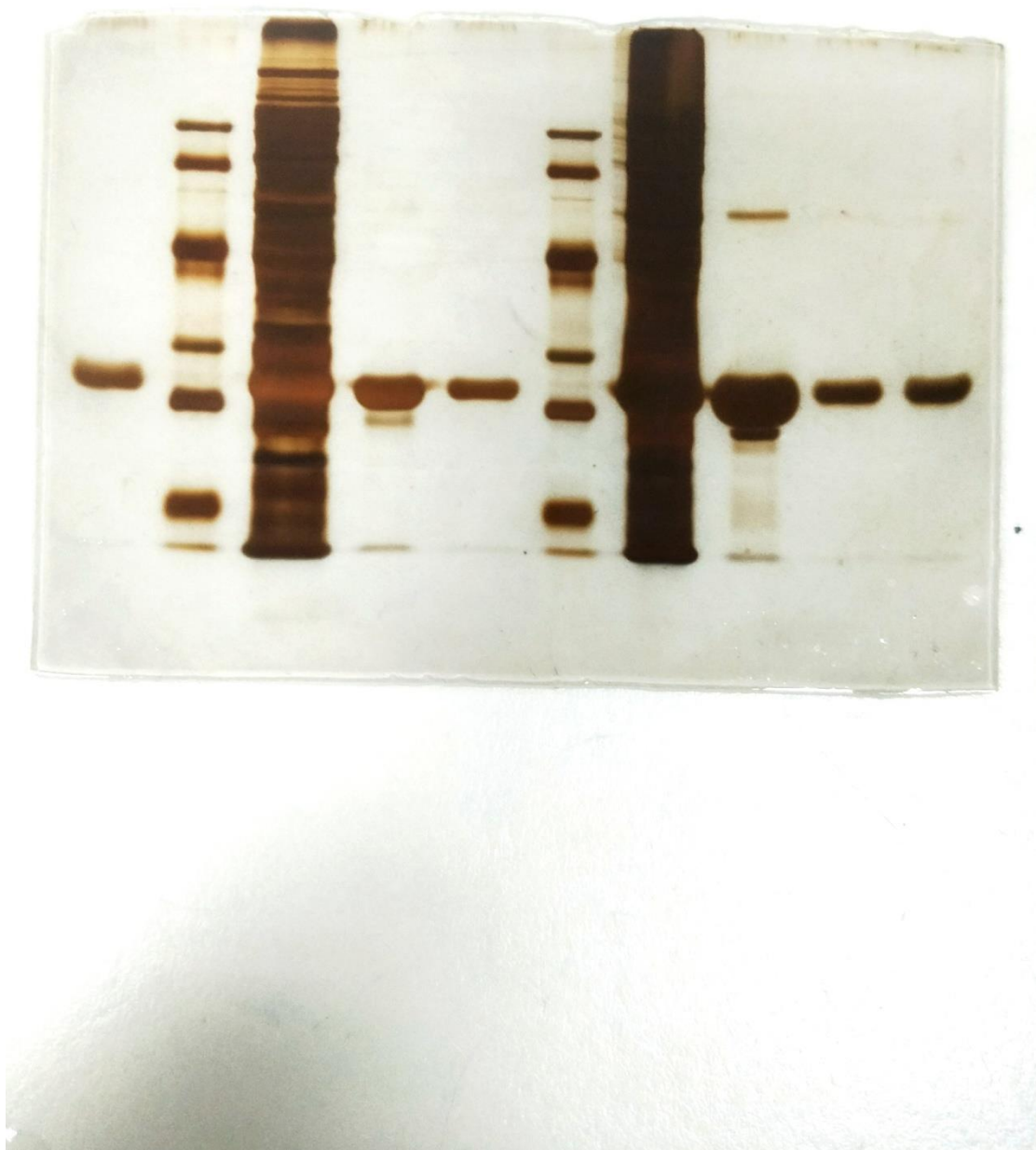

Figure 2 (g)

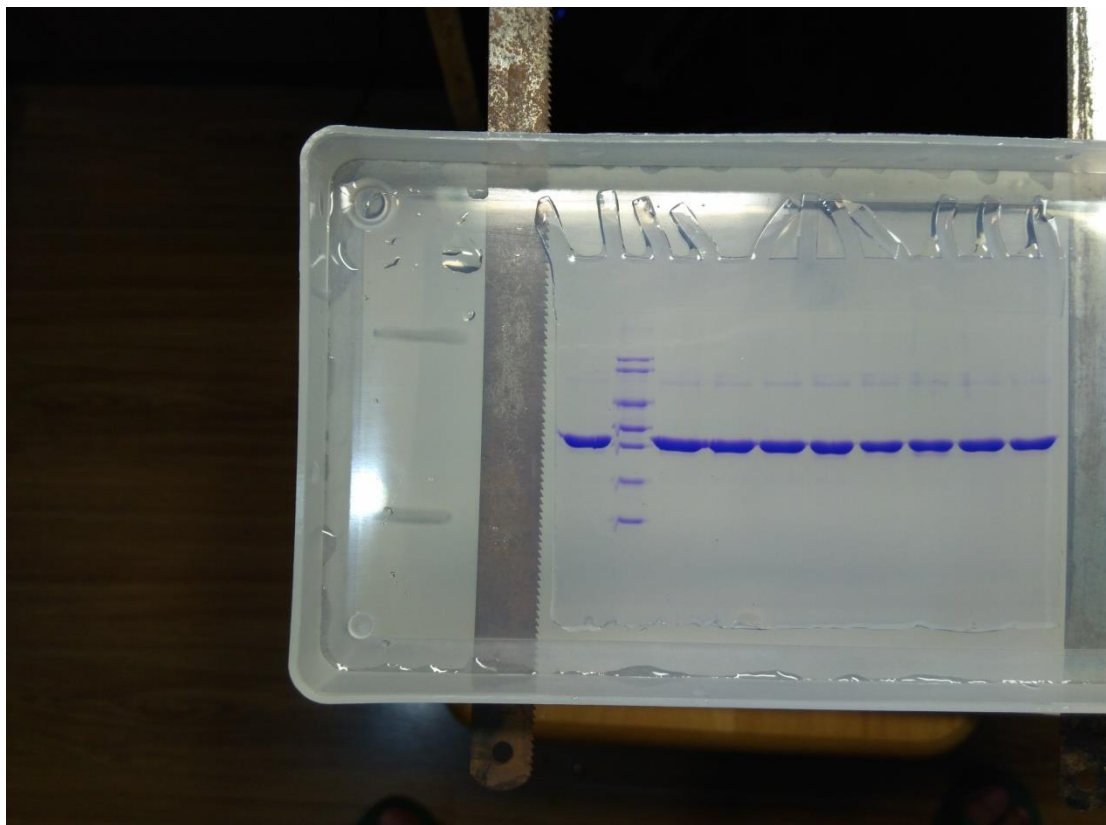

Figure 3 (a)

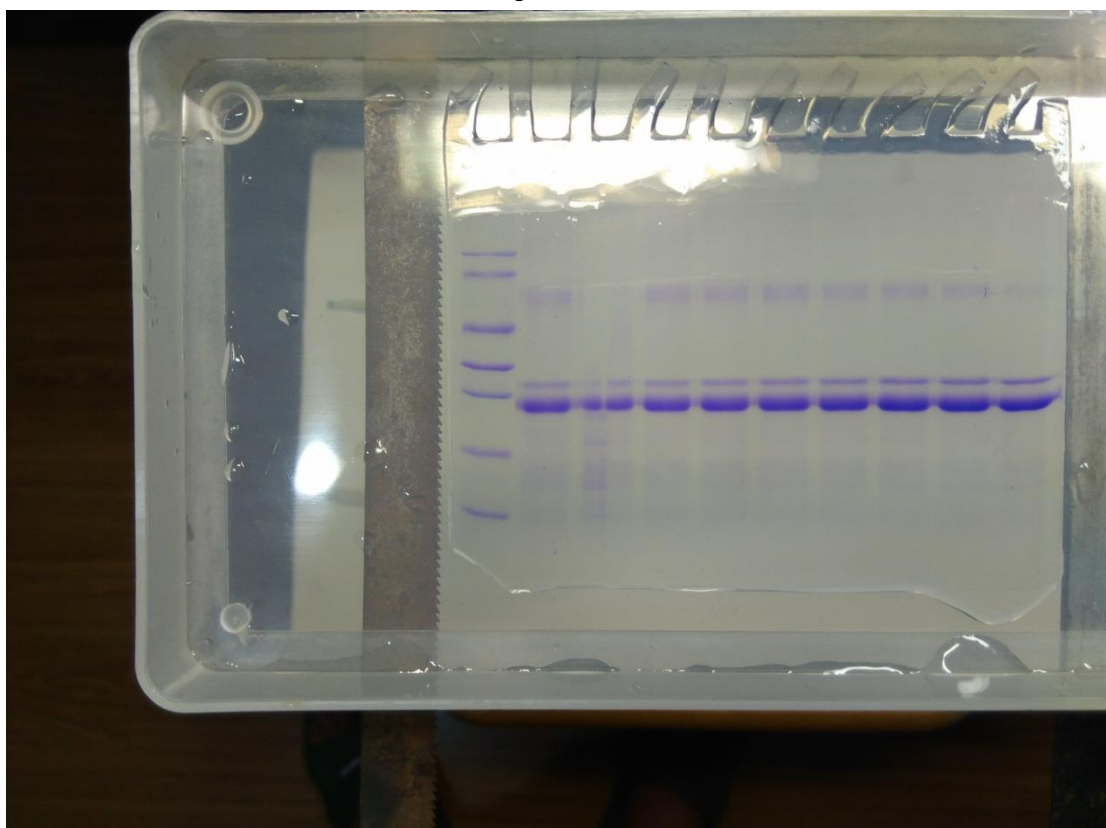

Figure 3 (b)

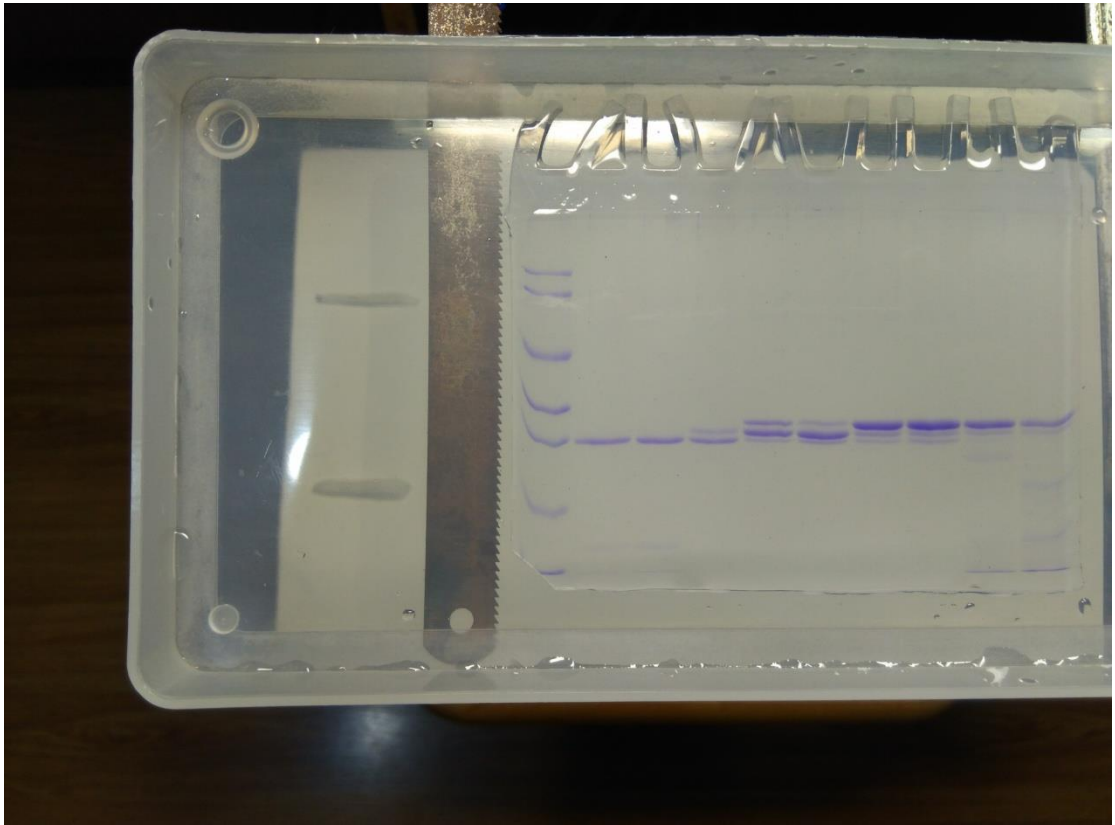

Figure 3 (c)

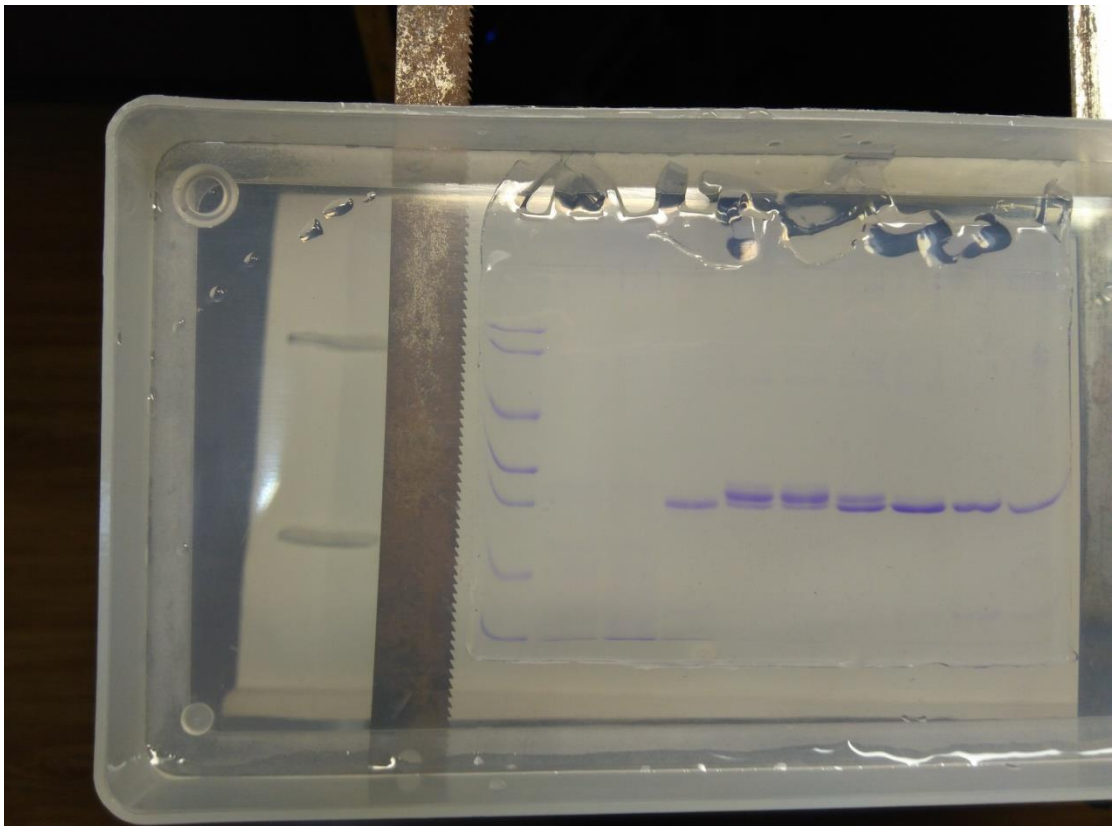

Figure 3 (d)

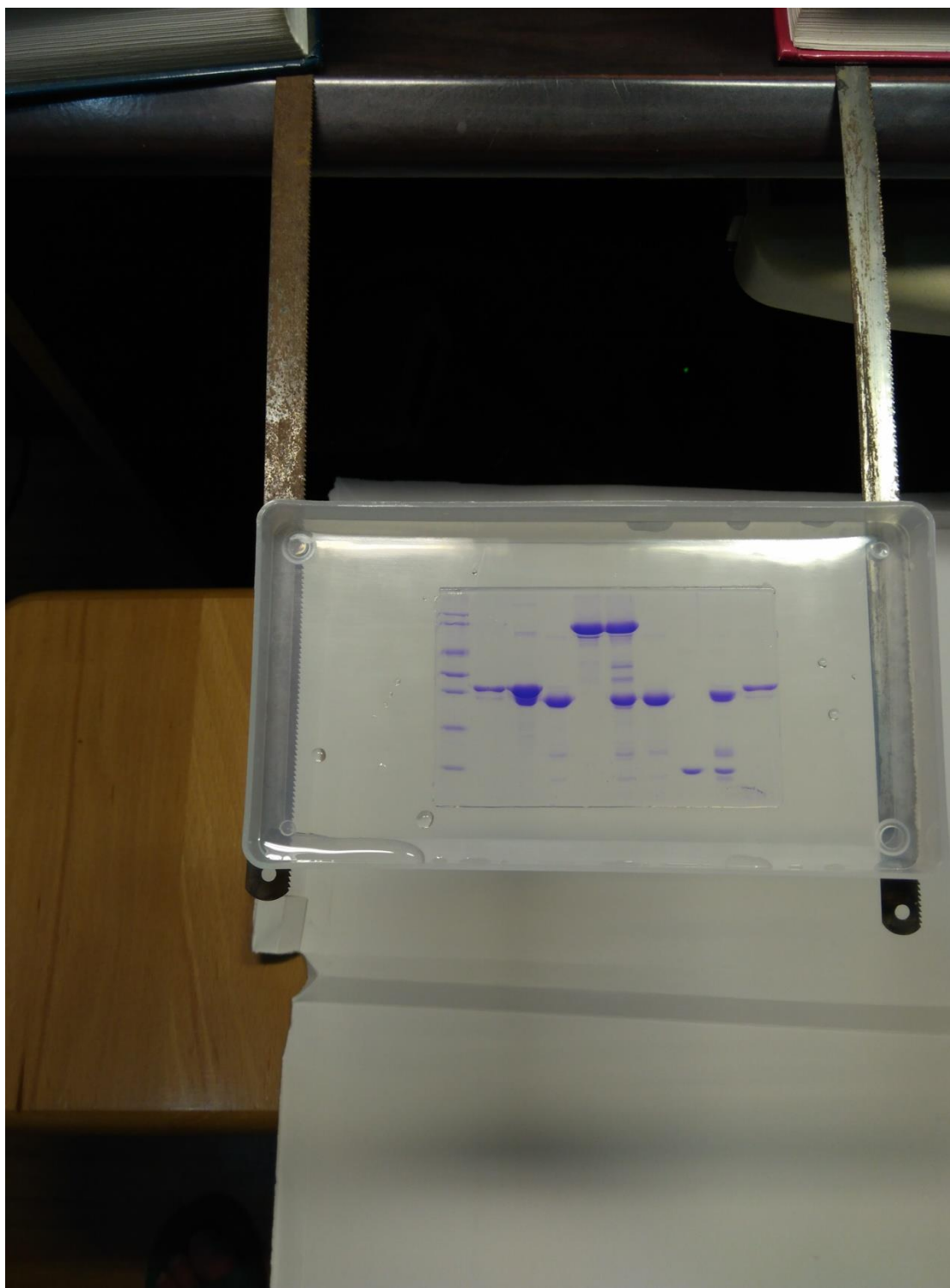

Figure 3 (e)

Supplement: Supplementary file 1 [file biomolecules-15-01357-s001.zip › biomolecules-3629684-File S1.Original image.pdf]
